# Hydralazine-associated adverse events: a report of two cases of hydralazine-induced ANCA vasculitis

**DOI:** 10.1590/2175-8239-JBN-3858

**Published:** 2018-05-07

**Authors:** Roman Zuckerman, Mayurkumar Patel, Eric J Costanzo, Harry Dounis, Rany Al Haj, Seyedehsara Seyedali, Arif Asif

**Affiliations:** 1 Jersey Shore University Medical Center, Neptune, NJ, USA.

**Keywords:** glomerulonephritis, lupus nephritis, glomerulosclerosis, focal segmental, glomerulonefrite, Lúpus, glomeruloesclerose, segmentar focal

## Abstract

Hydralazine is a direct-acting vasodilator, which has been used in treatment for hypertension (HTN) since the 1950s. While it is well known to cause drug-induced lupus (DIL), recent reports are indicating the emergence of the drug-induced anti-neutrophil cytoplasmic antibody (ANCA) associated vasculitis (DIV). Herein, we describe two patients (aged 57 and 87 years) who presented with severe acute kidney injury (AKI), proteinuria, and hematuria. Both were receiving hydralazine for the treatment of hypertension. ANCA serology was positive in both patients along with anti-histone antibodies (commonly seen in drug-induced vasculitis). Renal biopsy revealed classic crescentic (pauci-immune) glomerulonephritis in these patients and hydralazine was discontinued. During the hospital course, the 57-year-old patient required dialysis therapy and was treated with steroids and rituximab for the ANCA disease. Renal function improved and the patient was discharged (off dialysis) with a serum creatinine of 3.6 mg/dL (baseline = 0.9 mg/dL). At a follow-up of 2 years, the patient remained off dialysis with advanced chronic kidney disease (CKD) (stage IIIb). The 87-year-old patient had severe AKI with serum creatinine at 10.41 mg/dL (baseline = 2.27 mg/dL). The patient required hemodialysis and was treated with steroids, rituximab, and plasmapheresis. Unfortunately, the patient developed catheter-induced bacteremia and subsequently died of sepsis. Hydralazine can cause severe AKI resulting in CKD or death. Given this extremely unfavorable adverse-event profile and the widespread availability of alternative anti-hypertensive agents, the use of hydralazine should be carefully considered.

## RESUMO

A hidralazina é um vasodilatador de ação direta, que vem sendo utilizado no tratamento da hipertensão arterial (HA) desde a década de 1950. Embora seja bem conhecido por causar lúpus induzido por drogas (LID), relatórios recentes estão indicando o surgimento da vasculite associada ao anticorpo citoplasmático anti-neutrófilo (ANCA), induzida por drogas (VID). Aqui, descrevemos dois pacientes (com idade entre 57 e 87 anos) que apresentaram lesão renal aguda grave (LRA), proteinúria e hematúria. Ambos estavam usando hidralazina para o tratamento da hipertensão. A sorologia para ANCA foi positiva em ambos os pacientes, juntamente com anticorpos anti-histona (comumente vistos na vasculite induzida por drogas). A biópsia renal revelou glomerulonefrite rapidamente progressiva clássica (pauci-imune) nestes pacientes e a hidralazina foi interrompida. Durante a internação hospitalar, o paciente de 57 anos necessitou de diálise e foi tratado com esteroides e rituximab para a doença do ANCA. A função renal melhorou e o paciente recebeu alta (fora da diálise) com creatinina sérica de 3,6 mg/dL (basal = 0,9 mg/dL). Em um seguimento de 2 anos, o paciente permaneceu fora da diálise com doença renal crônica avançada (DRC) (estágio IIIb). O paciente de 87 anos apresentava IRA grave com creatinina sérica em 10,41 mg/dL (valor basal de = 2,27 mg/dL). O paciente necessitou de hemodiálise e foi tratado com esteroides, rituximabe e plasmaferese. Infelizmente, o paciente desenvolveu bacteremia induzida por cateter e, posteriormente, evoluiu a óbito por sepse. A hidralazina pode causar IRA grave, resultando em DRC ou óbito. Dado este perfil de eventos adversos extremamente desfavorável e a disponibilidade generalizada de agentes anti-hipertensivos alternativos, o uso de hidralazina deve ser considerado com muita parcimônia.

## Introduction

Hydralazine is a direct-acting vasodilator that has been used in treatment of hypertension since the 1950s. It also has been widely used in combination with beta blockers and diuretics in order to avoid reflex tachycardia and fluid retention respectively, associated with hydralazine therapy.^1^ Drug-induced lupus (DIL) associated with the use of hydralazine was first described in 1953.^2^ The incidence of hydralazine-induced lupus is 5-8%. The typical symptoms include arthralgia, myalgia, fever, rash, pleuritis, and leukopenia.^3^ Renal injury is uncommon, encountered in 5-10% of reported cases.^4^ However, cases demonstrating drug-induced vasculitis (DIV) limited to the kidneys associated with hydralazine use were reported in the literature beginning in the early 1980s.^5^^-^ 7 In this article, we present two cases of hydralazine-induced ANCA-associated vasculitis and raise the awareness of serious adverse events associated with hydralazine.

## Case Presentation

### Case 1

**Figure 1:**
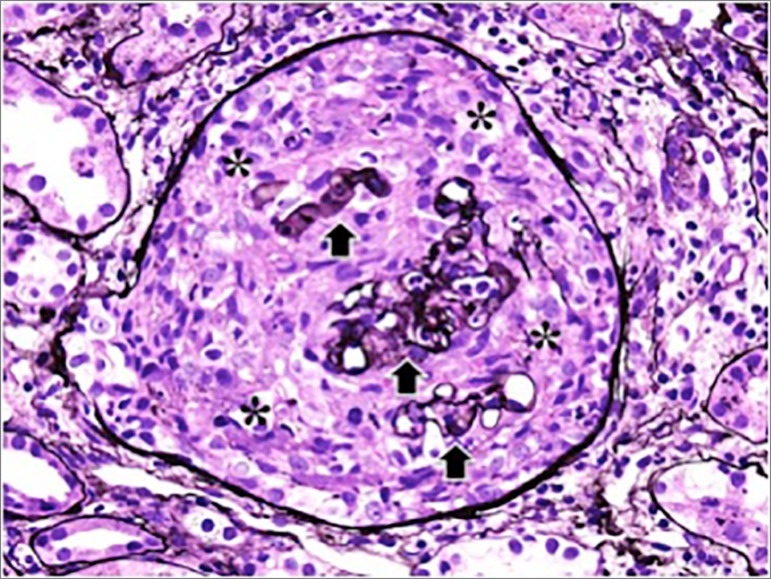
Crescentic glomerulonephritis. A glomerulus showing a circumferential cellular crescent (stars). Remnants of the glomerular tuft are demonstrated (arrows)

A 57-year-old Caucasian man with past medical history of hypertension and mild osteoarthritis, presented to the emergency department from the outpatient clinic with complaints of hematuria and acute renal failure (serum creatinine 3.6 mg/dL, baseline serum creatinine 0.9 mg/dL, six weeks before). Four days prior to the presentation, the patient was seen by the primary care for possible sinusitis, dysuria, and mild hematuria. Amoxicillin was prescribed for three days for a presumed urinary tract infection. The patient reported some fatigue, denied smoking or the use of alcohol and illicit drugs. Current medications included amlodipine 10 mg/day and hydralazine 50 mg BID, which was started six weeks before for better blood pressure control. There was no significant finding on the physical examination. The urinalysis revealed hematuria and low-grade proteinuria. Microscopic examination of the urine sediment revealed numerous dysmorphic red blood cells, several red blood cell casts, and occasional white blood cells. Renal ultrasound was normal. A diagnosis of hydralazine-induced DIV was considered and the medication was discontinued. Serology was positive for AHA, cANCA by immunofluorescence and PR3 by ELISA at 52 AU/mL, and an ANA titer at 1:1,115 with a homogenous pattern. Serum levels of C3 and C4 complements were normal. Antibodies to pANCA and MPO were not detected. Serology for anti-GBM, hepatitis panel, and HIV was negative. The patient was treated with high-dose pulse steroid therapy (500 mg/day for three days). However, the renal failure continued to progress (serum creatinine 4.0 mg/dL) and the patient required dialysis therapy due to hyperkalemia (K 5.6 mmol/L) and acidosis (serum bicarbonate 13). Kidney biopsy revealed pauci-immune necrotizing glomerulonephritis with increase in 20% of glomeruli (Figure 1). The diagnosis of hydralazine-induced DIV was made. The patient was treated with pulse steroid and rituximab. Renal function stabilized and dialysis was discontinued after four sessions. He was discharged on day twelve with normal electrolytes and serum creatinine of 3.4 mg/dL. PR3 and ANA were undetectable (Table 1). Two years later, the patient remained stable but with an advanced CKD stage III (serum Cr 2.8 mg/dL and eGFR 42). His blood pressure remained around 130-140/85-90 mmHg with amlodipine 10 mg/dL, chlorthalidone 12.5 mg/day, and ramipril 10 mg/day.

**Table 1:** Laboratory data of patients with hydralazine-induced ANCA associated vasculitis

	Case 1	Case 2
Laboratory values	57 y/o Caucasian male	87 y/o Caucasian male
Highest hydralazine dose	50 mg BID	100 mg TID
Duration of hydralazine therapy	6 weeks	5 years
BUN, mg/dL	33	102
Serum Cr, mg/dL	3.6	10.41
Baseline Cr, mg/dL	0.9	2.27
Urinalysis	Large blood/+1protein	Large blood/+1protein
ANCA titer/IF pattern	cANCA	1:160/pANCA
MPO titer, AU/mL	-	25
PR3 titer, AU/mL	52	-
Anti-Chromatin abs (normal < 19 Units)	Not checked	31
ANA titer/IF pattern	1:1,115/homogeneous	1:640/homogeneous
Anti-dsDNA	-	+
Anti-dsDNA by Crithidia	-	1:160
AHA IgG (normal < 0.9 Units)	8.7	3.1
C3 (normal 85 - 170 mg/dL)	88	50.4
C4 (normal 16 - 40 mg/dL)	22.8	11.1
ESR, (normal < 20 mm/hour)	39	41
Outcome	CKD Stage IIIb	Death secondary to catheter-associated sepsis

### Case 2

**Figure 2:**
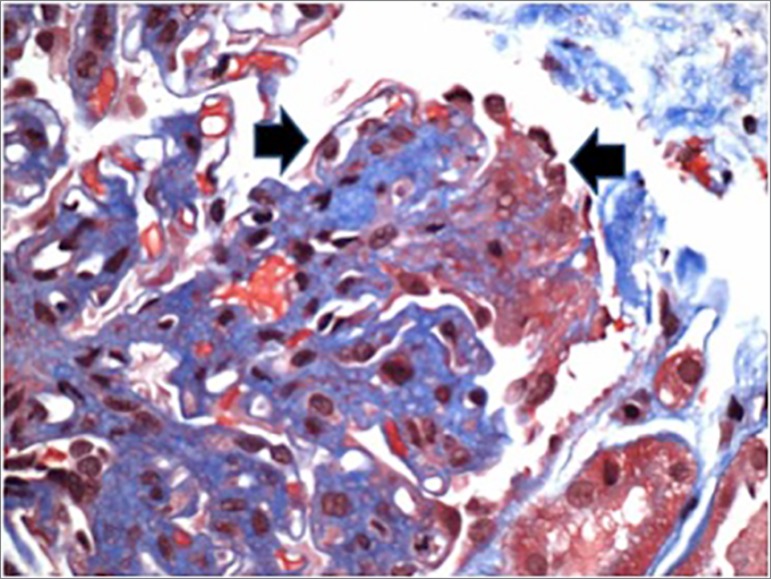
Glomerulus with fibrocellular crescent (arrow)

An 87-year-old Caucasian man with past medical history of hypertension (HTN), dementia, and CKD III presented to the hospital with altered mental status and AKI. There was no report of fever, chills, dysuria, hematuria, rashes, arthralgia or myalgia. His medications included hydralazine, isosorbide mononitrate, furosemide, doxazosin, atorvastatin, aspirin, duloxetine, and pantoprazole. The patient was on hydralazine for a total of five years with the most recent dose of 100 mg thrice per day (TID), increased from 50 mg TID three years ago. There was no significant finding on the physical examination. The laboratory work revealed serum Cr 10.41 mg/dL and BUN 102 mg/dL (baseline 2.27 and 42 one year before). Urinalysis showed hematuria and +1 proteinuria. Protein/Cr ratio was 3.1 gm and ESR 41 mm/hr. Serology was positive for pANCA by immunofluorescence at 1:160, MPO by ELISA at 25 AU/mL, AHA at 3.1 units, anti-chromatin antibodies at 31 U, ANA titer at 1:640 with homogeneous pattern, positive dsDNA by ELISA and crithidia at 1:160, C3 at 50.4 mg/dL, and C4 at 11.1 mg/dL. Serology for anti-GBM, cANCA/PR3, and hepatitis panel was all negative (Table 1). Renal ultrasound was normal. Patient was started on emergency hemodialysis. Hydralazine-induced DIV was suspected given positive vasculitis serologic workup. The patient received a pulse dose steroid course and then started on plasmapheresis. Subsequently, he underwent a kidney biopsy, which showed pauci-immune focal crescentic glomerulonephritis confirming the diagnosis (Figure 2). Unfortunately, the kidney function did not show signs of recovery and rituximab therapy was initiated. After receiving two doses, however, further treatment was held due to dialysis catheter-induced bacteremia. Due to the rapidly deteriorating mental status and overall condition of the patient, the family decided to withdraw care and transfer the patient to hospice service.

## Discussion

The clinical presentation of hydralazine-induced DIV is usually more severe than that of DIL. It usually involves the skin, with involvement of kidneys and lungs also.^8^ Patients can present with rapidly progressing necrotizing and crescentic glomerulonephritis along with arthralgia, upper airway involvement, pulmonary disease, and cutaneous vasculitic findings.^9^ The two cases presented here underscore the importance of serious adverse events (i.e. the development of severe AKI and subsequent CKD and death) associated with hydralazine-induced ANCA vasculitis. Clinically, this disease resembles the idiopathic ANCA-associated vasculitis (AAV), such as granulomatosis with polyangiitis and microscopic polyangiitis.^10^ Serologies reveal high ANCA titer to MPO with characteristic pANCA staining pattern, anti-nuclear antibodies (ANA), anti-histone antibodies (AHA), as well as “atypical” ANCAs.^8^ The ANCAs, which target lactoferrin and human leukocyte elastase, were found to be strongly associated with hydralazine-induced DIV.^11^ Both cases reported here were found to be positive for anti-histone antibodies. The case number 2 was positive for pANCA with an MPO titer of 25 AU/mL. Our case number 1 was positive for cANCA with PR3 titer of 52 AU/mL. Several reports showed positivity for cANCA and PR3 antibodies in propylthiouracil-induced DIV.^12^^-^ 14 However, a previously reported case of hydralazine-induced DIV by Agarwal et al. was positive for PR3 but also for MPO antibodies.^15^ In this context, our case number 1 is the first reported case of hydralazine-induced isolated cANCA, PR3 positive vasculitis. In addition, our patient developed advanced CKD (stage IIIb) following acute kidney injury, while in Agarwal’s case the renal function recovered, with serum creatinine returning to 1.1 mg/dL compared to 2.13 mg/dL at the time of admission.

The risk factors for developing DIV associated with hydralazine use include longer duration of therapy and especially higher daily and cumulative doses^16^^,^ 17 The acetylator status appears to be a strong predictive factor for developing the disease, with slow acetylators having decreased hepatic synthesis of N-acetyltransferase.^18^^,^ 19 DNA methylation is essential for regulation of gene expression and T-cell function.^20^ DNA hypomethylation is responsible for activation of lymphocyte-associated antigen 1 (LFA-1) (CD11a/CD18) gene transcription and induction of auto-reactivity in SLE.^21^^-^ 23 In a similar fashion, hydralazine has shown to inhibit DNA methylation by affecting the DNA methyltransferase (DNMT) through the inhibition of the extracellular signal-regulated kinase (ERK) pathway.^24^ Similar to patients with idiopathic AAV, DNA hypomethylation in hydralazine-induced DIV might be responsible for disrupting the silencing of PR3 and MPO. This increases autoantigen expression in neutrophils.^9^^,^ 25 ^,^ 26 In a study evaluating the antineoplastic potential of hydralazine, it acted as a non-nucleoside DNA methylation inhibitor, reversing epigenetic silencing of tumor suppressor genes.^27^

Establishing the diagnosis of drug-induced vasculitis (DIV) in a patient with multiple comorbidities who initially presents with renal failure is a challenge for a clinician. Kidney failure secondary to diseases like diabetes and hypertension can potentially mask and delay the diagnosis of DIV. In the absence of other organ involvement, especially the skin, the diagnosis relies on the positive serologies and renal histopathology. Discontinuing hydralazine is the first step in treatment and this alone might be sufficient; however, more aggressive management involving immunosuppression is often necessary. The available options include steroids, cyclophosphamide, rituximab, and plasmapheresis. The therapeutic approach varies based on patient’s comorbidities, severity of the renal injury, organ involvement, and age.

The choice of hydralazine in the management of HTN has largely been replaced by newer antihypertensive drugs with more acceptable side effect profiles.^1^ According to the Eighth Joint National Committee (JNC8), hydralazine is not recommended as a first line therapy for HTN, since there was no good or fair quality randomized control trials comparing the four recommended drug classes.^28^ However, it is still widely used in pregnant patients and heart failure patients, especially in developing countries due to its low cost.^1^ The addition of a hydralazine-nitrate combination to a standard therapy in African-American patients with heart failure and reduced ejection fraction has shown to reduce mortality by 43% as well as number of hospitalizations for heart failure and length of hospital stay.^29^^,^ 30 Nevertheless, given an extremely unfavorable side effect profile and multiple alternatives available on the market, hydralazine should generally be avoided. In situations where its use is necessary due to either unavailability of other agents, intolerance or inefficiency it is imperative for the clinician to monitor closely the patients on a long-term/high dose hydralazine regimen.

## Conclusion

While drug-induced lupus has been frequently reported with the use of hydralazine, this medication can also cause drug-induced ANCA vasculitis. The two cases presented here provide evidence of the detrimental adverse effects of hydralazine including acute kidney injury leading to chronic kidney disease and even death.
